# A national survey of 'inactive' physicians in the United States of America: enticements to reentry

**DOI:** 10.1186/1478-4491-9-7

**Published:** 2011-02-17

**Authors:** Ethan A Jewett, Sarah E Brotherton, Holly Ruch-Ross

**Affiliations:** 1 Division of Workforce and Medical Education Policy, American Academy of Pediatrics, Elk Grove Village, IL, USA; 2 Dept of Data Acquisition Services, American Medical Association, 515 N State St., Chicago, IL 60654, USA; 3 Independent Research Consultant, Evanston, IL, USA

## Abstract

**Background:**

Physicians leaving and reentering clinical practice can have significant medical workforce implications. We surveyed inactive physicians younger than typical retirement age to determine their reasons for clinical inactivity and what barriers, real or perceived, there were to reentry into the medical workforce.

**Methods:**

A random sample of 4975 inactive physicians aged under 65 years was drawn from the Physician Masterfile of the American Medical Association in 2008. Physicians were mailed a survey about activity in medicine and perceived barriers to reentry. Chi-square statistics were used for significance tests of the association between categorical variables and t-tests were used to test differences between means.

**Results:**

Our adjusted response rate was 36.1%. Respondents were fully retired (37.5%), not currently active in medicine (43.0%) or now active (reentered, 19.4%). Nearly half (49.5%) were in or had practiced primary care. Personal health was the top reason for leaving for fully retired physicians (37.8%) or those not currently active in medicine (37.8%) and the second highest reason for physicians who had reentered (28.8%). For reentered (47.8%) and inactive (51.5%) physicians, the primary reason for returning or considering returning to practice was the availability of part-time work or flexible scheduling. Retired and currently inactive physicians used similar strategies to explore reentry, and 83% of both groups thought it would be difficult; among those who had reentered practice, 35.9% reported it was difficult to reenter. Retraining was uncommon for this group (37.5%).

**Conclusion:**

Availability of part-time work and flexible scheduling have a strong influence on decisions to leave or reenter clinical practice. Lack of retraining before reentry raises questions about patient safety and the clinical competence of reentered physicians.

## Background

Physician reentry first achieved recognition as an important workforce policy issue in 2002, with an article by Mark et al. in which physician reentry was defined as "returning, after an extended absence, to the professional activity/clinical practice for which one has been trained, certified or licensed" [1]. Discussions within the United States of America began among federal policy makers, medical and specialty societies, and educators, leading to the American Academy of Pediatrics (AAP) establishing a multi-organizational Physician Reentry into the Workforce Project (Reentry Project) in 2006. In 2008, the AAP and the American Medical Association (AMA) co-sponsored the Physician Reentry to the Workforce Conference to identify steps for the implementation of a formal physician reentry system. Both the Reentry Project and the AMA have produced a number of resources that examine issues related to physician reentry [2-4].

Very little data on physician reentry exist. A state-level study by Rimsza in Arizona and a survey of physicians over age 50 by the Association of American Medical Colleges (AAMC) and several specialty societies have provided some important data [5-7]. In addition, Freed et al. conducted studies on clinical inactivity among pediatricians and state medical board licensure policies for active and inactive physicians, reporting that 5% of pediatricians were currently inactive, and 12% had at some point experienced a period of clinical inactivity of 12 months or more [8,9]. Because of numerous data gaps identified by the AAP Reentry Project, a survey was fielded in early 2008 on physician reentry into the workforce.

## Methods

A questionnaire (see Additional File 1) was developed using an iterative process with input from members of the AAP Reentry Project Workforce Workgroup and others with expertise in physician workforce issues. Questions were based on those used in the AAMC Survey of Physicians Over 50, conducted in 2006. The Physician Workforce Reentry questionnaire included separate sets of questions for physicians not currently active in medicine and those currently active in medicine. The latter were asked about their experiences leaving and reentering the workforce. Areas of inquiry included reasons for not being active in medicine, planning and experiences related to becoming active again, and several demographic questions.

The questionnaire, with a post-paid return envelope, was mailed to a random sample of 4975 out of 14 113 inactive physicians under the age of 65 years drawn from the Physician Masterfile of the American Medical Association (AMA). The Physician Masterfile is a repository of current and historical information on over 1 million physicians in the United States. The Masterfile is used for AMA membership purposes (although not all physicians in the Masterfile are AMA members) as well as for medical credentials verification, and thus keeping the information current is an ongoing activity. The 'inactive' category in the Masterfile includes individuals who work less than 20 hours per week and report that they are retired, semi-retired, temporarily not in practice or not active for other reasons ('active' physicians are those who report being in direct patient care, or in medical education, research, administration or other medical activities, and work more than 20 hours total per week in those activities). Physicians living outside of the United States were not included in the sample. Respondents were offered a small incentive for prompt return of the questionnaire at each of three rounds (a drawing for gift certificates) in January, February and March 2008.

Data were analyzed using the Statistical Package for the Social Sciences, v. 16. A chi-square statistic was used to test for the significance of the association between categorical variables in contingency tables. T-tests were used to test the significance of differences between means. The Institutional Review Board of the AAP judged this study exempt.

## Results

After three mailings, a total of 1576 completed surveys were returned. Another 613 surveys were returned marked "deceased" or with bad addresses. The adjusted response rate was 36.1% (1576/4362). Females (42.2%, vs. 32.8% for males, *P *< 0.001), those over age 60 (38.4%, vs. 34.6% for under 60, *P *< 0.01), and those with addresses in the Midwest or West of the United States (40.3% Midwest; 39.8% West; 34.5% South; 30.1% Northeast; *P *< 0.001) all had somewhat elevated response rates.

Respondents were asked, "Are you currently active in medicine?" and were provided examples of activity in medicine (providing clinical services, conducting medical research, medical teaching, health-care administration, and other professional medical activities). Responses that could be selected were: currently active in medicine; fully retired from medicine; not currently active in medicine; and never active in medicine. Although members of the sample were identified as "inactive" at last entry into the Masterfile, 584 (37.0%) reported they were currently active in medicine at the time of our survey, and of these, 358 reported that they had not taken a leave from medicine of 6 months or more. These latter respondents may have been among those who were coded as "inactive" because they had indicated they were semi-retired, or temporarily not in practice at the time of their last AMA census response but may have been working in, for example, medical education (although fewer than 20 hours per week). We excluded them from the analysis, as, for our purposes, they had never been not active in medicine. We included the remaining 226 currently active respondents who reported that they had at some point taken a leave of six months or more from active medicine, and had then reentered medicine. Nine respondents were excluded because they reported they had never been active in medicine, and 47 were excluded for failing to answer the screening question, "Are you currently active in medicine?" This left a final sample of 1162 physicians, divided into three groups: 436 (37.5%) fully retired, 226 (19.4%) reentered, and 500 (43.0%) not currently active.

Table 1 reports characteristics of respondents by status. As expected, the fully retired group was older than both of the other two groups. This group also included the lowest proportion of females. Respondents were predominantly married (77.8%), white (86.2%) and of non-Hispanic ethnicity (95.8%). The reentered group was more likely to report excellent or very good health status (75.6% vs. 58.9%, retired, and 59.3%, inactive). The reentered and fully retired groups reported somewhat better financial health than those not currently active. There were no significant differences between the groups for location of medical school (89.4% United States) or for board certification rate (36.5%) (data not shown). The fully retired group had proportionately more general surgeons and physicians in other surgical specialties, while the reentered group had more internists, and the not currently active group had more pediatricians.

**Table 1:** Characteristics of fully retired, reentered and not currently active respondents

	Fully Retired	Reentered	Not currently active	All respondents
	(n = 436)	(n = 226)	(n = 500)	(n = 1162)
Age, mean, yrs ^a^	60.1	54.9	55.4	57.1
	% (n)	% (n)	% (n)	% (n)

Gender ^a^				
Female	31.6 (137)	50.4 (114)	4938 (248)	43.1 (499)
Male	68.4 (296)	49.6 (112)	50.2 (250)	56.9 (658)

Marital status				
Married/partnered	80.5 (347)	78.2 (176)	75.2 (373)	77.8 (896)
Divorced/separated	10.2 (44)	12.4 (28)	11.1 (55)	11.0 (127)
Widowed	1.9 (8)	1.8 (4)	3.2 (16)	2.4 (28)
Single	7.4 (32)	7.6 (17)	10.5 (52)	8.8 (101)

Race				
White	88.9 (378)	86.8 (191)	87.5 (426)	86.2 (1276)
Asian	4.5 (19)	6.4 (14)	5.1 (25)	5.1 (58)
All others	6.6 (28)	6.8 (15)	7.4 (36)	7.0 (79)

Hispanic origin				
Yes	4.5 (19)	3.2 (7)	4.3 (21)	4.2 (47)

Overall health status ^a^				
Excellent	35.3 (151)	38.2 (86)	35.6 (177)	36.0 (414)
Very good	23.6 (101)	37.3 (84)	23.7 (118)	26.3 (303)
Good	17.5 (75)	17.3 (39)	20.7 (103)	18.9 (217)
Fair	18.2 (78)	6.2 (14)	13.9 (69)	14.0 (161)
Poor	5.4 (23)	0.9 (2)	6.0 (30)	4.8 (55)

Current financial status ^a^				
Excellent	29.8 (127)	29.5 (66)	25.2 (124)	27.7 (317)
Very good	30.3 (129)	28.6 (64)	24.3 (120)	27.4 (313)
Good	25.4 (108)	25.0 (56)	29.0 (143)	26.9 (307)
Fair	13.1 (56)	13.4 (30)	13.2 (65)	13.2 (151)
Poor	1.4 (6)	3.6 (8)	8.3 (41)	4.8 (55)

Primary specialty/subspecialty ^a^				
Family medicine	15.0 (57)	17.1 (36)	17.6 (79)	16.5 (172)
Pediatrics	8.7 (33)	7.1 (15)	14.0 (63)	10.7 (111)
Internal medicine	9.5 (36)	20.0 (42)	13.6 (61)	13.4 (139)
Ob-gyn	10.8 (41)	7.6 (16)	8.0 (36)	8.9 (93)
General surgery	7.1 (27)	1.4 (3)	3.1 (14)	4.2 (44)
Other medical specialty	29.5 (112)	36.2 (76)	32.7 (147)	32.2 (335)
Other surgical specialty	19.5 (74)	10.5 (22)	11.1 (50)	14.0 (146)

^a ^*P *< 0.001

Table 2 reflects the current experience and status of respondents not currently in the workforce. Over half of those who are fully retired (59.9%) or currently inactive (62.4%) reported last being active in medicine five or more years previously. More of the not currently active group (27.1%) are currently working in non-medical fields than of the fully retired group (16.9%), but substantial majorities of both groups did not report working in another field. The majority (71.2%) of those who are fully retired reported they have no future plans to become active in medicine; of those not currently active in medicine, 55.3% were "not sure" about plans to return. A large majority of both groups reported retaining at least some medical licenses, although the fully retired respondents were somewhat more likely to report that they had not retained any licensure. Among those with specialty or subspecialty certification, similar majorities reported that their certifications were current. Only a minority had retained any medical liability insurance, and this was almost always tail coverage only. Fully retired respondents were slightly more likely to report retaining tail coverage.

**Table 2:** Physicians who are fully retired or not currently active in medicine (N = 936)

	Fully retired (n = 436)	Not currently active (n = 500)
	**% (n)**	**% (n)**

How long since last active in medicine ^a^		
Less than 1 year	3.2 (14)	6.7 (33)
1-2 years	15.9 (69)	11.8 (58)
3-4 years	21.0 (91)	19.1 (94)
5-10 years	38.7 (168)	38.3 (189)
More than 10 years	21.2 (92)	24.1 (119)
*missing*	(2)	(7)
Currently working in other field ^b^		
Yes	16.9 (73)	27.1 (135)
*missing*	(4)	(1)

Plan to become active in future ^b^		
Yes, within a year	1.9 (8)	11.1 (55)
Yes, in one to five years	0.9 (4)	11.5 (57)
Yes, more than five years from now	0.2 (1)	1.0 (5)
No	71.2 (307)	21.1 (105)
Not sure	25.8 (111)	55.3 (275)
*missing*	(5)	(3)

Retained medical licenses ^b^		
Yes, all of them	47.1 (204)	59.6 (297)
Yes, but not all	19.9 (86)	20.5 (102)
No	33.0 (143)	19.9 (99)
*missing*	(3)	(2)

Specialty/subspecialty board certification(s) current		
Yes, all of them	56.1 (242)	54.9 (272)
Yes, but not all	3.0 (13)	5.3 (26)
No	27.1 (117)	24.4 (121)
Not certified	13.7 (59)	15.4 (76)
*missing*	(5)	(5)

Retained medical liability insurance ^a^		
Yes, tail coverage only	31.6 (136)	24.5 (121)
Yes, full liability coverage	1.6 (7)	2.0 (10)
No	65.6 (282)	69.6 (344)
Other	1.2 (5)	3.8 (19)
*missing*	(6)	(6)

^a ^*P *<.05

^b ^*P *<.001

Those who have reentered active medicine reported a mean of 40.6 hours worked per week. Among these respondents, the average length of time they had been away from active medicine was 4.3 years (not shown).

Table 3 reports the reasons that respondents retired or became inactive. The most frequently cited reason for being fully retired or not currently active in medicine was personal health issues (37.8% for both groups); this reason was frequently cited among those who had reentered active medicine as well (28.8%), second only to the need to care for young children (29.6%). Substantial proportions of both fully retired (27.8%) and not currently active (21.4%) physicians cited rising medical malpractice premiums as a reason for leaving active medicine; this was the reason for a substantially smaller proportion of those who had reentered (13.7%). Fully retired physicians were more likely to cite 'hassle factors' (37.4%) and insufficient reimbursement (20.6%) as reasons for leaving medicine. Those not currently active were more likely than the other physicians to cite the need to care for other family members (15.2%).

**Table 3:** Reasons not currently active or reason became inactive (before reentry) ^a^

	Fully retired (n = 436)	Not currently active (n = 500)	Reentered (n = 226)
	**Reason not currently active**		**Reason was inactive **^**b**^

	**% (n)**	**% (n)**	**% (n)**

Personal health issues/concerns	37.8 (165)	37.8 (189)	28.8 (65)
"Hassle factor" (ex: paperwork, compliance issues)^d^	37.4 (163)	28.2 (141)	21.7 (49)
Rising medical malpractice premiums ^c^	27.8 (121)	21.4 (107)	13.7 (31)
Lack of professional satisfaction	25.2 (110)	22.2 (111)	19.9 (45)
On call responsibility	19.0 (83)	17.6 (88)	11.9 (27)
Insufficient reimbursement rates ^c^	20.6 (90)	15.0 (75)	14.6 (33)
Pursuing a non-medical career ^c^	12.2 (53)	17.8 (89)	6.6 (15)
Need to care for young children ^e^	6.4 (28)	18.4 (92)	29.6 (67)
Practice not economically viable	12.8 (56)	11.8 (59)	10.6 (24)
Improvement in personal/family finances	13.1 (57)	9.2 (46)	7.1 (16)
Need to care for other family member(s) ^e^	5.5 (24)	15.2 (76)	6.6 (15)
Hard to keep up with clinical advances	5.5 (24)	5.0 (25)	0.4 (1)
Inadequate practice volume	2.8 (12)	2.0 (10)	0.4 (1)
Other	24.5 (107)	9.4 (147)	32.3 (73)

^a ^Positive responses; multiple response permitted

^b ^No statistics testing of reentered vs. other groups (questions are different)

^c ^*P *< 0.05, fully retired vs. not currently active

^d ^*P *< 0.01, fully retired vs. not currently active

^e ^*P *< 0.001, fully retired vs. not currently active

Reasons for becoming active again are shown in Table 4. Responses were significantly different between those who were fully retired and those who were not currently active; the leading response among the former group (34.2%) was that "nothing" would lead them to consider becoming active in medicine again. However, when we exclude those who responded that "nothing" would lead them to consider returning to active medicine, the appeal of many of the remaining reasons to return was very similar for the two groups. The most common response among those not currently active was that availability of part-time work or flexible scheduling (51.1%) would lead them to consider becoming active in medicine again; this was also a common, but less frequent, response among those who were fully retired (42.5%, *P *< 0.05). The availability of part-time work or flexible scheduling was also, by far, the most commonly cited reason for becoming active again among those who had reentered (47.8%).

**Table 4:** Reasons to consider becoming active in medicine again or reason reentered ^a^

	Fully retired (n = 436)		Not currently active (n = 500)		Reentered (n = 226)	
	Reasons to consider reentry				**Reasons for Reentry **^**b**^	
	%	(n)	%	(n)		
Nothing ^e^	34.2	(149)	3.6	(18)		
Reasons among those who did not indicate "nothing" would lead them to consider reentry	(n = 287)		(n = 482)			

	%	(n)	%	(n)	%	(n)

Availability of part-time work or flexible scheduling ^c^	42.5	(122)	51.5	(248)	47.8	(108)
Financial need	43.9	(126)	43.4	(209)	32.3	(73)
Desire to provide volunteer services	40.8	(117)	39.6	(191)	8.0	(18)
Change in family or personal circumstances ^e^	30.1	(89)	42.9	(207)	31.0	(70)
Responding to a need in the community	33.1	(95)	38.0	(183)	16.8	(38)
Miss caring for patients ^c^	29.3	(84)	37.3	(180)	32.7	(74)
Miss colleagues/practice environment	19.9	(57)	23.4	(113)	22.6	(51)
Want to pursue a new challenge or new area of medicine ^e^	10.5	(30)	21.0	(101)	16.8	(38)
Boredom/Too much free time on my hands	12.9	(37)	17.6	(85)	13.3	(30)
An opportunity to change my specialty/subspecialty with relative ease ^d^	8.0	(23)	15.6	(75)	9.7	(22)
An opportunity with less administrative responsibility	7.3	(21)	8.3	(40)	10.6	(24)
Other	22.0	(63)	25.7	(124)	19.0	(43)

^a ^Positive responses; multiple response permitted

^b ^No statistical testing of reentered vs. other groups (questions are different).

^c ^*P *< 0.05, fully retired vs. not currently active

^d ^*P *< 0.01, fully retired vs. not currently active

^e ^*P *< 0.001, fully retired vs. not currently active

Nearly a quarter (23.7%) of the fully retired respondents had explored becoming active in medicine again; respondents who were not currently active were twice as likely (50.3%) to report having explored returning to medicine (Table 5). Both groups had used similar strategies to explore reentry, and over 80% of both groups felt that it would be difficult. Of those who had reentered active medicine, slightly more than a third (35.9%) reported that it was difficult to reenter. All three groups were likely to identify limited opportunities for part-time or flexible work schedules as a barrier to reentry. Only 37.5% of the reentered group had retraining before entering practice again. Those who had retraining were, on average, out of the workforce significantly longer than those who did not (6.1 years vs. 2.9 years, F = 28.56, *P *< 0.001; not shown). Very few of those who reported receiving retraining had been involved in what might be described as formal training for reentry; seven had been in a reentry program, and five were in mini-residencies. Many more used continuing medical education, either online (15.9%) or live (22.1%), as their reentry educational program.

**Table 5:** Efforts to reenter active medicine, not currently active and reentered physicians (n = 1162)

	Fully retired (n = 436)		Not currently active (n = 500)		Reentered (n = 226)	
	**%**	**(n)**	**%**	**(n)**	**%**	**(n)**

Ever explored becoming active in medicine again ^a^						
Yes	23.7	(101)	50.3	(237)		n/a
*missing*		(9)		(23)		

How explored becoming active in medicine ^b ^(n = 341)						
Did some reading about the process or requirements	28.7	(29)	38.3	(92)		n/a
Talked to professional colleagues	51.5	(52)	45.8	(110)		n/a
Contacted state about licensing	25.7	(26)	27.9	(67)		n/a
Contacted Specialty Board about recertification ^c^	2.0	(2)	9.2	(22)		n/a
Contacted a medical liability insurance company regarding a new policy	8.9	(9)	13.8	(33)		n/a
Talked to potential employers	41.6	(42)	40.4	(97)		n/a
Contacted medical school	12.9	(13)	7.5	(18)		n/a
Other	27.7	(28)	22.9	(55)		n/a

Easy or difficult to reenter medicine						
Easy	17.0	(16)	16.5	(36)	64.1	(141)
Difficult	83.0	(78)	83.5	(182)	35.9	(79)

Barriers identified ^b ^(n = 341)						
State licensure requirements	28.7	(29)	30.0	(72)	17.7	(40)
Specialty Board recertification requirements	10.9	(11)	15.4	(37)	3.8	(22)
Insurance company requirements	29.7	(30)	26.7	(64)	22.1	(50)
Employer requirements	20.8	(21)	20.4	(49)	13.3	(30)
Restrictions on hospital privileges	14.9	(15)	20.8	(50)	11.9	(27)
Limited opportunities for retraining	31.7	(32)	40.4	(97)	15.9	(36)
Cost of retraining	27.7	(28)	23.8	(57)	8.4	(19)
Limited opportunities for part-time or flexible work hours	44.6	(45)	42.5	(102)	26.1	(59)
Family constraints	10.9	(11)	16.7	(40)	10.6	(24)
Other barriers	26.7	(27)	35.0	(84)		
No barriers		n/a		n/a	32.3	(73)

Had retraining before reentering medicine						
Yes		n/a		n/a	37.5	(84)
Retraining experience						
Formal reentry program		n/a		n/a	3.1	(7)
Mini-residency		n/a		n/a	2.2	(5)
Federal Medical Reserve Corps		n/a		n/a	0	(0)
Shadowing an active physician		n/a		n/a	10.6	(24)
Online continuing medical education		n/a		n/a	15.9	(36)
Live continuing medical education		n/a		n/a	22.1	(50)
Other		n/a		n/a	15.5	(35)

^a ^*P *< 0.001, fully retired vs. not currently active

^b ^positive responses; multiple response permitted

^c ^*P *< 0.05, fully retired vs. not currently active

### Gender analysis

Additional analyses were performed to examine possible gender differences in family and work responsibilities of our respondents. Table 6 presents the reasons for leaving active medicine for those not currently active and those who have reentered active medicine. Among those not currently active, the most striking differences are the much higher proportions of women who indicate the need to care for young children (35.5% vs. 1.6%, *P *< 0.001) or for other family members (23.4% vs. 7.2%, *P *< 0.001) as to why they left active practice. Among those who have reentered active practice, men are more likely to report reasons for leaving related to the structure and practice of medicine ('hassle factor', malpractice premiums, lack of professional satisfaction, insufficient reimbursement, practice not viable) and women to report family needs (care for young children, care for other family members). Overall, characteristics of the practice environment were cited infrequently as a reason for leaving among women who have reentered, especially in comparison to men of either group, but also compared to women who are currently inactive.

**Table 6:** Reasons left active medicine for those not currently active and those who have reentered, by gender ^a^

	**Not currently active **^**b**^				Reentered			
	**Female (n = 248)**		**Male (n = 250)**		**Female (n = 114)**		**Male (n = 112)**	

	**%**	**(n)**	**%**	**(n)**	**%**	**(n)**	**%**	**(n)**

Personal health issues/concerns	34.3	(85)	41.2	(103)	26.3	(30)	31.3	(35)
'Hassle factor' (ex: paperwork, compliance issues)	27.8	(69)	28.8	(72)	13.2 ^d^	(15)	30.4	(34)
Rising medical malpractice premiums	19.8	(49)	23.2	(58)	3.5 ^e^	(4)	24.1	(27)
Lack of professional satisfaction	21.8	(54)	22.8	(57)	13.2 ^d^	(15)	26.8	(30)
On-call responsibility	19.4	(48)	16.0	(40)	7.9	(9)	16.1	(18)
Insufficient reimbursement rates	13.7	(34)	16.4	(41)	6.1 ^e^	(7)	23.2	(26)
Pursuing a non-medical career	14.1^c^	(35)	21.6	(54)	4.4	(5)	8.9	(10)
Need to care for young children	35.5^e^	(88)	1.6	(4)	56.1 ^e^	(64)	2.7	(3)
Practice not economically viable	13.3	(33)	10.4	(26)	4.4 ^d^	(5)	17.0	(19)
Improvement in personal/family finances	9.7	(24)	8.8	(22)	5.3	(6)	8.9	(10)
Need to care for other family member(s)	23.4 ^e^	(58)	7.2	(18)	10.5 ^c^	(12)	2.7	(3)
Hard to keep up with clinical advances	7.7^d^	(19)	2.4	(6)	0.9	(1)	0	
Inadequate practice volume	0.8	(2)	3.2	(8)	0		0.9	(1)
Other	25.8	(64)	32.4	(81)	25.4 ^c^	(29)	39.3	(44)

^a ^Positive responses; multiple response permitted

^b ^Two physicians not currently active in medicine did not report their gender.

^c ^*P *< 0.05, female vs. male within activity group

^d ^*P *< 0.01, female vs. male within activity group

^e ^*P *< 0.001, female vs. male within activity group

Both female and male physicians who are not currently active in medicine report diverse reasons that might lead them to consider becoming active in medicine again (Table 7). Women were significantly more likely than men to report availability of part-time work or flexible scheduling (57.7% vs. 41.6%, *P *< 0.001) and a change in family or personal circumstances (53.2% vs. 30.0%, *P *< 0.001) as reasons to consider becoming active again. However, among those who have reentered, missing colleagues is also a reason more likely to be reported by female respondents (28.1% vs. 17.0%, *P *< 0.05). Men were significantly likely to report reentering to pursue a new challenge (24.1% vs. 9.6%, *P *< 0.001) or an opportunity with less administrative responsibility (16.1% vs. 5.3%, *P *< 0.01).

**Table 7:** Reasons to reenter active medicine, by gender ^a^

	**Not Currently Active **^**b**^				Reentered			
	**Reasons to consider becoming** active in medicine again				Reasons reentered active medicine			
	**Female** **(N = 248)**		**Male** **(N = 250)**		**Female** **(N = 114)**		**Male** **(n = 112)**	

	**%**	**(n)**	**%**	**(n)**	**%**	**(n)**	**%**	**(n)**

Availability of part-time work or flexible scheduling	57.7^e^	(143)	41.6	(104)	54.4^c^	(62)	41.1	(46)
Financial need	42.7	(106)	41.2	(103)	28.1	(32)	36.6	(41)
Desire to provide volunteer services	41.5	(103)	35.2	(88)	7.9	(9)	8.0	(9)
Change in family or personal circumstances	53.2^e^	(132)	30.0	(75)	43.9^e^	(50)	17.9	(20)
Responding to a need in the community	35.9	(89)	37.6	(94)	12.3	(14)	21.4	(24)
Miss caring for patients	37.1	(92)	34.8	(87)	37.7	(43)	27.7	(31)
Miss colleagues/practice environment	23.4	(58)	22.0	(55)	28.1^c^	(32)	17.0	(19)
Want to pursue a new challenge or new area of medicine	23.4	(58)	16.8	(42)	9.6 ^d^	(11)	24.1	(27)
Boredom/Too much free time on my hands	17.7	(44)	16.4	(41)	12.3	(14)	14.3	(16)
An opportunity to change my specialty/subspecialty with relative ease	21.0^e^	(52)	9.6	(24)	11.4	(13)	8.0	(9)
An opportunity with less administrative responsibility	5.6	(4)	10.0	(25)	5.3 ^d^	(6)	16.1	(18)
Other	23.4	(58)	26.8	(67)	14.9	(17)	23.2	(26)
Nothing	2.8	(7)	4.0	(10)	n/a		n/a	

^a ^Positive responses; multiple response permitted

^b ^Two physicians not currently active in medicine did not report their gender.

^c ^*P *< 0.05, male vs. female, within workforce status

^d ^*P *< 0.01, male vs. female, within workforce status

^e ^*P *< 0.001, male vs. female, within workforce status

## Discussion

Concerns have been raised over the last several years about a current or impending physician workforce shortage within the United States [10-12]. The potential of inactive or retired physicians to fill a workforce gap has not yet been adequately explored. The cost of mobilizing this 'shadow workforce' of physicians, either in a long-term capacity or to respond to an acute health emergency (e.g. a bioterrorist attack, pandemic, or natural disaster), is likely to be significantly less than that of expanding medical school class sizes and residency training slots. It would also be more efficient, as the timeframe for a reentry training program (variable from program to program) is substantially shorter than for training new physicians from scratch. Reincorporating these physicians into the active workforce would allow the public to benefit from their clinical knowledge and experience and recuperate its financial investment in the initial training of these physicians.

In this study of inactive physicians younger than age 65, the average length of time away from medicine for reentered physicians was 4.3 years. However, over 60% of the currently inactive and retired physicians had been out of medicine 5 or more years, including a fifth to a quarter for more than 10 years. Less than a quarter of currently inactive physicians had firm plans to reenter. Over two thirds of retired physicians and 80% of inactive physicians kept at least one medical license, although this may be relatively easy to achieve as there are few states that require measures of clinical activity to maintain licensure [9].

Given the amount of time out of practice for some of these physicians, formal training in any reentry pathway, if so chosen, is critical. In the last 10 years, major developments in pharmacology, surgical procedures, medical technology, coding, patient privacy, quality improvement--to name just a few--have dramatically altered practice. Increasing demands from the public for documentation of competence will have to be addressed, particularly considering only 37.5% of reentered physicians reported having any retraining before returning to practice. Freed et al. found that pediatricians who had been clinically inactive were less likely compared to those who had been continuously active to agree that a formal reentry program be required after an absence of 2 years [8]. Although this could be the result of over-confidence in one's ability, this could also reflect the difficulty of finding accessible programs. Formal reentry programs are few, and often present financial and geographical barriers, and may likely account for the low incidence of use among survey respondents. Live and online continuing medical education (CME) will, therefore, need to target the learning needs of inactive and reentering physicians and prepare them to face the challenges of a quickly evolving practice environment. An individualized plan to maintain professional credentials and relationships during inactivity, moreover, may help physicians who are thinking of leaving the workforce for an extended period to anticipate needs for CME, licensure, board certification, credentialing, networking, and other areas, so that they will be able to return to practice more easily.

A common perception among inactive physicians is that reentry to practice would be difficult. The actual experience may not be so, as a majority of respondents who had reentered did not find the process difficult. Easy access to information on how to return to practice, as well as guidance on how to maintain professional credentials during inactivity, may help to dispel the misconceptions of retired and inactive physicians. Free-response answers on the survey suggest that some inactive physicians perceive the health care system to be too complicated and inflexible to permit them to reenter.

The influence of family responsibilities on the decision to withdraw from clinical practice was particularly felt by female physicians in our study, as found by others [8]. The ability to work part-time or with a flexible schedule was the reason most often cited for being able to reenter by those women who had, and was the most compelling factor that would lead currently inactive women to reenter. The same is true for male physicians, who more often stated they left clinical practice for personal health reasons. The importance of a reduced or flexible schedule for these physicians cannot be overstated. A full one quarter of inactive physicians is working in fields other than medicine, which may be the result of their dissatisfaction with the structure of the current health care system. The 'hassle factor' of practice, rising malpractice premiums, insufficient reimbursement, and professional dissatisfaction were frequently cited by retired and inactive physicians as reasons they left medicine; many of them are now working in areas that, presumably, do not have these negative characteristics. Fewer reentered physicians cited these characteristics as reasons they had initially left medicine. Physicians who choose to return may not have experienced as intensely the hassles of practice--thus their return--or alternatively, have rationalized their return by 'softening' the negative memories of their past practice experience. These physicians are working, on average, 40.6 hours a week, which for many physicians would be a part-time schedule. Such a practice arrangement may serve to reduce the 'pain' of the perceived 'hassles' of the past, and it is clearly more accommodating for those with conflicting family responsibilities. Addressing these structural issues would likely reduce the number of physicians who choose to become inactive in the first place.

Our response rate of 36.1% was low, yet not surprising. Our population of physicians - 'inactives' in the AMA's Physician Masterfile - conjures up a cohort of physicians not highly engaged in medicine, with a matching lack of interest in a survey about their inactivity. In addition, over 20% of initial respondents considered themselves active in medicine and had not taken a leave from medicine longer than 6 months, suggesting that there is room for interpretation as to what an inactive physician actually is. We do not generalize our findings to all inactive physicians, who are most likely a particularly nebulous group. We do hope that we have provided a useful start at describing a group of physicians who could be encouraged to stay active in the workforce.

## Conclusions

Looking to the future, stakeholders in a stable and robust physician workforce will need to foster flexibility in the health care system, create incentives for physicians to return to practice, and develop resources to facilitate the reentry into the medical workforce. Survey respondents in all categories identified needed improvements in a number of areas, ranging from regulatory requirements--such as state licensure, insurance companies, and employers--to the cost and availability of retraining opportunities and limited opportunities for part-time work and flexible scheduling. It is tempting to speculate on how many of these physicians would have stayed active if part-time or flexible work hours had been available either in practice or in residency. Strategies to retain physicians will, therefore, need to account for the changing demographics of the physician population and their priority to balance their professional and personal lives. Finally, the development and promotion of better educational resources for physicians, especially those that would allow doctors to maintain their professional credentials and access affordable and relevant CME, would enable more predictable departures and reentry. A coordinated and comprehensive agenda that includes educational, research, regulatory and public policy efforts will thus be required to overcome barriers to physician reentry into the medical workforce and to respond effectively to national workforce needs.

## Competing interests

The authors declare that they have no competing interests.

## Authors' contributions

EAJ was principal investigator and acquired the funding. EAJ and HRR designed the survey. SEB and HRR acquired the data. HRR analyzed the data and all three authors interpreted the data, wrote the manuscript, and approved the final version.

## Supplementary Material

Additional file 1 **Physician workforce survey**. Click here for file
