# Supplementary material for: A national survey of 'inactive' physicians in the United States of America: enticements to reentry
Source: Hum Resour Health. 2011 Feb 17;9:7. doi: 10.1186/1478-4491-9-7 (PMC3050793; doi:10.1186/1478-4491-9-7)
Supplement: Additional file 1 — Physician workforce survey. [file 1478-4491-9-7-S1.PDF]

PHYSICIAN WORKFORCE SURVEY

This questionnaire is designed to obtain information from physicians regarding current and future practice plans. All responses will be kept strictly confidential and presented only in aggregate.

Please answer the questions by placing an X in the appropriate box, by circling the appropriate number, or by filling in the blanks. Please mark only ONE response unless requested to do otherwise.

> *Please note*: On this survey, **active in medicine** includes providing clinical services, conducting medical research, medical teaching, health-care administration and other professional medical activities.

1. Are you currently active in medicine?

Yes, currently active in medicine	☐	® ***SKIP** to Q11*	B. Currently Active in Medicine
No, fully retired from medicine	☐	® ***GO** to Q2*	A. Not Currently Active in Medicine
No, not currently active in medicine	☐	® ***GO** to Q2*	A. Not Currently Active in Medicine
Never active in medicine	☐	® ***SKIP** to Q18*	C. Background Information

A. PHYSICIANS NOT CURRENTLY ACTIVE IN MEDICINE

2. Approximately how long has it been since you were last active in medicine? ("Active in medicine" includes providing clinical services, conducting medical research, medical teaching, health-care administration and other professional medical activities.)

☐ Less than 1 year
☐ 1-2 years
☐ 3-4 years
☐ 5-10 years
☐ More than 10 years

3. What are the reasons that you are not currently active in medicine? *(Please check all that apply)*

☐ Pursuing a non-medical career
☐ Practice not economically viable
☐ Personal health issues/concerns
☐ Need to care for young children
☐ Need to care for other family member(s)
☐ On call responsibility
☐ Insufficient reimbursement rates
☐ Rising medical malpractice premiums
☐ Lack of professional satisfaction
☐ Improvement in personal/family finances
☐ Inadequate practice volume
☐ Hard to keep up with clinical advances
☐ "Hassle factor" (ex: paperwork, compliance issues)
☐ Other, specify: ___________________________

4. Are you currently working for pay in a field other than medicine?

☐ No
☐ Yes → **Please describe:**____

5. Do you plan to become active in medicine in the future?

- ☐ Yes, within a year
- ☐ Yes, in one to five years
- ☐ Yes, more than five years from now
- ☐ No
- ☐ Not sure

6. Have you maintained your medical license(s), including full, restricted and inactive licensure?

- ☐ Yes, all of them
- ☐ Yes, but not all
- ☐ No

7. Is/are your specialty/subspecialty board certification(s) current?

- ☐ Yes, all of them
- ☐ Yes, but not all
- ☐ No
- ☐ Not certified

8. Have you retained any type of medical liability insurance?

- ☐ Yes tail coverage only
- ☐ Yes, full liability coverage
- ☐ No
- ☐ Other *please explain* ______________________________

9. What might lead you to consider becoming active in medicine again? *(Please check all that apply)*

- ☐ Change in family or personal circumstances
- ☐ Financial need
- ☐ An opportunity with less administrative responsibility
- ☐ Availability of part-time work or flexible scheduling
- ☐ Boredom/Too much free time on my hands
- ☐ Miss colleagues/practice environment
- ☐ Responding to a need in the community
- ☐ Miss caring for patients
- ☐ An opportunity to change my specialty/subspecialty with relative ease
- ☐ Want to pursue a new challenge or new area of medicine
- ☐ Desire to provide volunteer services
- ☐ Nothing
- ☐ Other (please specify) ______________________________

10. **Have you ever explored the possibility of becoming active in medicine again?**

- ☐ No
- ☐ Yes ® **a. What did you do to explore this possibility?** (*Please check all that apply)*
  - ☐ Did some reading about the process or requirements
  - ☐ Talked to professional colleagues
  - ☐ Contacted state about licensing
  - ☐ Contacted Specialty Board about recertification
  - ☐ Contacted a medical liability insurance company regarding a new policy
  - ☐ Talked to potential employers
  - ☐ Contacted medical school
  - ☐ Other *please describe* ____

  b. Did you find that it would be easy or difficult to reenter medicine?

  ☐ Easy ☐ Difficult

  c. What barriers did you identify? (Please c*heck all that apply)*
  - ☐ State licensure requirements
  - ☐ Specialty Board recertification requirements
  - ☐ Insurance company requirements
  - ☐ Employer requirements
  - ☐ Restrictions on hospital privileges
  - ☐ Limited opportunities for retraining
  - ☐ Cost of retraining
  - ☐ Limited opportunities for part-time or flexible work hours
  - ☐ Family constraints
  - ☐ Other barriers *please describe* ____

Please go to Question 18 (Section C) if you are not currently active in medicine.

B. PHYSICIANS CURRENTLY ACTIVE IN MEDICINE

If you are active in medicine, please answer Questions 11 through 17.

11. **During your most recent complete week of work, approximately how many hours were you active in medicine** (including providing clinical services, conducting medical research, medical teaching, health-care administration and other professional medical activities. Please include on-call time while you were actively engaged in patient care or coordination)?

_____________ hours/week

12. **Have you ever taken an extended (six months or more) leave of absence from medicine?**

Note: Please do not include an academic sabbatical.

- ☐ No ® **SKIP** to Q18
- ☐ Yes ® ***Please answer the following questions (through 17) in relation to your most recent period of absence from medicine.***

  How long were you away from medicine?________________year(s) _____________month(s)

13. What are the reasons that you left active medicine? *(Please check all that apply)*

- ☐ Pursuing a non-medical career
- ☐ Practice not economically viable
- ☐ Personal health issues/concerns
- ☐ Need to care for young children
- ☐ Need to care for other family member(s)
- ☐ On call responsibility
- ☐ Insufficient reimbursement rates
- ☐ Rising medical malpractice premiums
- ☐ Lack of professional satisfaction
- ☐ Improvement in personal/family finances
- ☐ Inadequate practice volume
- ☐ Hard to keep up with clinical advances
- ☐ "Hassle factor" (ex: paperwork, compliance issues)
- ☐ Other, specify: ___________________________

14. What were the reasons you reentered active medicine? *(Please check all that apply)*

- ☐ Change in family or personal circumstances
- ☐ Financial need
- ☐ An opportunity with less administrative responsibility
- ☐ Availability of part-time work or flexible scheduling
- ☐ Boredom/Too much free time on my hands
- ☐ Missed colleagues/practice environment
- ☐ Responded to a need in the community
- ☐ Missed caring for patients
- ☐ An opportunity to change my specialty/subspecialty with relative ease
- ☐ Wanted to pursue a new challenge or new area of medicine
- ☐ Desire to provide volunteer services
- ☐ Other, specify

15. Did you find it easy or difficult to reenter?

☐ Easy ☐ Difficult

16. What barriers did you encounter? (Please c*heck all that apply)*

- ☐ State licensure requirements
- ☐ Specialty Board recertification requirements
- ☐ Insurance company requirements
- ☐ Employer requirements
- ☐ Restrictions on hospital privileges
- ☐ Limited opportunities for retraining
- ☐ Cost of retraining
- ☐ Limited opportunities for part-time or flexible work hours
- ☐ Family constraints
- ☐ No barriers

17. Did you have any retraining before reentering medicine?

☐ No

☐ Yes → **Did your retraining experience include any of the following?** *(Please check all that apply)*

- ☐ Formal reentry program
- ☐ Mini-residency
- ☐ Federal Medical Reserve Corps
- ☐ Shadowing an active physician
- ☐ Online continuing medical education
- ☐ Live continuing medical education
- ☐ Other, *please specify* ____

C. BACKGROUND INFORMATION

Regardless of your status in medicine, we would greatly appreciate it if you would answer Questions 18 through 29.

18. Year of birth: ___________

19. Gender:

☐ Female ☐ Male

20. Marital Status:

☐ Married/Partnered
☐ Divorced/Separated
☐ Widowed
☐ Single

21. For how many individuals are you financially responsible?

Under age 21	Age 21 or older
☐ None	☐ None
☐ One	☐ One
☐ 2-3	☐ 2-3
☐ 4 or more	☐ 4 or more

22. Race:

☐ Asian or Pacific Islander
☐ Black/African American
☐ Native American/Alaskan
☐ White
☐ Multiple Races
☐ Other, specify:__________________________

23. Are you of Hispanic origin?

☐ Yes ☐ No

24. How would you rate your overall health status?

☐ Excellent
☐ Very good
☐ Good
☐ Fair
☐ Poor

25. How would you rate your current financial status?

☐ Excellent
☐ Very good
☐ Good
☐ Fair
☐ Poor

26. Please provide the information on your medical education:

a. Year of graduation from medical school: _______

b. Location of medical school:

- ☐ United States
- ☐ Canada
- ☐ Other Country → Specify: ______________________________

27. When did you complete your most recent graduate medical training (residency, fellowship):

__________________ (year)

28. Do you have any other degrees? *(Check all that apply)*

- ☐ PhD
- ☐ MPH
- ☐ MBA
- ☐ JD
- ☐ MA/MS/MEd
- ☐ Other (Describe): ______________________

29. What best describes your primary specialty/subspecialty (specialty in which you currently spend or have spent the most hours weekly) and secondary specialty/subspecialty?

**Specialty/subspecialty**	**Primary** (check if yes)	**Secondary** (check if yes)	**Certified** (check if yes)
Family medicine	☐	☐	☐
Pediatrics	☐	☐	☐
Internal Medicine	☐	☐	☐
Obstetrics-Gynecology	☐	☐	☐
General Surgery	☐	☐	☐
Other medical specialty	☐	☐	☐
Other surgical specialty	☐	☐	☐

THANK YOU FOR YOUR HELP!

Please return this survey in the enclosed envelope to: Physician Workforce Survey, c/o Data Shop, Inc., P.O. Box 5203, Janesville, WI 53547-5203

The Physician Reentry into the Workforce Project

Physician reentry into practice can be defined as returning to professional activity/clinical practice for which one has been trained, certified or licensed after an extended time period. This is an issue that cuts across genders and specialties. However, anecdotal evidence indicates that reentry into the workforce will affect women more often than men.

There are many reasons why physicians leave medicine and then seek to reenter the workforce. These include the need to care for both children and older parents; personal illness; the changing demands of academic and community practice; professional dissatisfaction; and alternative careers.

Although there is a paucity of data on this complex topic, many agree that it is an issue that is gaining in prominence. Some of the key considerations that individuals, organizations and institutions face include:

- Ensuring that there is an adequate and well-trained physician workforce
- Having the ability to rapidly bring physicians back into the workforce to meet public health needs in a national emergency
- Maintenance of Board Certification requirements
- State Medical Boards' licensure requirements
- Reestablishing hospital privileges and/or credentials
- Demonstrating competency to institutions and/or peers
- Obtaining appropriate retraining
- Establishing guidelines or criteria to determine what types of retraining an individual might need
- Identifying funding resources in an era of budget cuts
- Providing guidance and resources to society members

The American Academy of Pediatrics (AAP) Division of Graduate Medical Education & Pediatric Workforce is spearheading the "Physician Reentry into the Workforce Project." The AAP, in collaboration with the American Medical Association Women Physicians Congress (AMA-WPC) and the organizations listed below, is in the process of developing an agenda that will guide our future actions. The agenda will enable us to continue to move forward as a group to learn more and share ideas and experiences. While each organization will need to address its specific needs, if we are all operating within the same framework, we can better identify areas for collaboration and ultimately create guidelines or recommendations that will serve all of our organizations and our members.
